# Frequency of dengue virus–specific T cells is related to infection outcome in endemic settings

**DOI:** 10.1172/jci.insight.179771

**Published:** 2025-02-24

**Authors:** Rosa Isela Gálvez, Amparo Martínez-Pérez, E. Alexandar Escarrega, Tulika Singh, José Victor Zambrana, Ángel Balmaseda, Eva Harris, Daniela Weiskopf

**Affiliations:** 1 Center for Vaccine Innovation, La Jolla Institute for Immunology, La Jolla, California, USA.; 2 Division of Infectious Diseases and Vaccinology, School of Public Health, University of California, Berkeley, Berkeley, California, USA.; 3 Sustainable Sciences Institute, Managua, Nicaragua.; 4 Department of Epidemiology, School of Public Health, University of Michigan, Ann Arbor, Michigan, USA.; 5 Laboratorio Nacional de Virología, Centro Nacional de Diagnóstico y Referencia, Ministerio de Salud, Managua, Nicaragua.; 6 Division of Infectious Diseases and Global Public Health, School of Medicine, University of California, San Diego, La Jolla, California, USA.

**Keywords:** Immunology, Infectious disease, Vaccines, Adaptive immunity, T cells

## Abstract

Dengue is widespread in tropical and subtropical regions globally and imposes a considerable disease burden. Annually, dengue virus (DENV) causes up to 400 million infections, of which approximately 25% present with clinical manifestations ranging from mild to fatal. Despite its significance as a growing public health concern, developing effective DENV vaccines has been challenging. One reason is the lack of comprehensive understanding of the influence exerted by prior DENV infections and immune responses with cross-reactive properties. To investigate this, we collected samples from a pediatric cohort study in dengue-endemic Managua, Nicaragua. We characterized T cell responses in 71 healthy children who had previously experienced 1 or more natural DENV infections and who, within 1 year after sample collection, had a subsequent DENV infection that was either symptomatic or inapparent. Our study investigated the effect of preexisting DENV-specific T cell responses on clinical outcomes of subsequent DENV infection. We assessed DENV-specific T cell responses using an activation-induced marker assay. Children with only 1 prior DENV infection displayed heterogeneous DENV-specific CD4^+^ and CD8^+^ T cell frequencies. In contrast, children with 2 or more prior DENV infections showed significantly higher DENV-specific CD4^+^ and CD8^+^ T cell frequencies associated with inapparent rather than symptomatic outcomes in subsequent infection. These findings demonstrate the protective role of DENV-specific T cells against symptomatic DENV infection and advance efforts to identify protective immune correlates against dengue.

## Abstract 2

Dengue, a widespread tropical disease causing up to 400 million infections annually, lacks effective vaccines. Our study in a Nicaraguan children cohort reveals that 2 prior dengue infections are needed to have Dengue-specific CD8^+^ T cells that are able to protect against a subsequent symptomatic infection.

## Graphical abstract

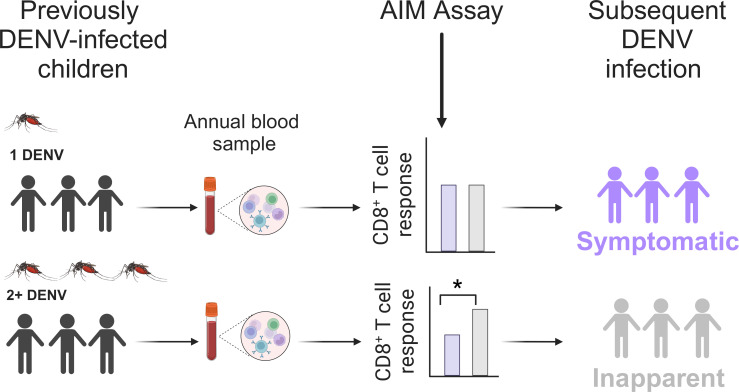

## Introduction

In 2023, the WHO declared dengue to be the world’s fastest-spreading tropical disease and to represent a “pandemic threat” to nearly half of the global population (1). Dengue is caused by 4 dengue virus (DENV) serotypes, which are enveloped viruses containing a single-stranded, positive-sense RNA genome (2). DENVs circulate worldwide in the tropics and subtropics and annually cause an estimated 400 million DENV infections, with up to 100 million resulting in clinical cases and at least 22,000 deaths (3). Understanding the sequential development of immunity to DENVs is crucial since individuals living in endemic regions are sequentially exposed to multiple serotypes. The precise protective and immunopathogenic mechanisms related to sequential DENV infections with different serotypes than those responsible for the original infection (heterologous infection) remain only partially elucidated (4). Substantial evidence suggests that the humoral arm of the adaptive immune response, triggered by the initial infection, plays a role in both protecting against disease and enhancing DENV replication during subsequent infection (5–7). Cohort studies have demonstrated that, in cases of secondary heterologous DENV infection, the duration of viremia and, more importantly, infection and disease outcome are influenced by the level of preexisting antibodies against DENV, such that a specific range of antibody titers is associated with the likelihood of protection or the risk of experiencing severe dengue during a subsequent infection (8). Interestingly, this relation appears to depend on the serotype of the subsequent infection (7, 9). The underlying distinct mechanisms are yet to be defined.

In a similar manner to the humoral response, the adaptive cellular responses exerted by T cells are acknowledged to play a pivotal role in the control of DENV infections (4, 10, 11). T cells provide durable protective immunity (12), yet they have also been linked to eliciting immune-mediated damage (13). However, the precise factors governing the establishment of memory T cell responses in the context of multiple DENV infections are still incompletely understood. The presence of preexisting T cell memory has the potential to significantly influence outcome during secondary heterologous DENV infection, promoting faster and more robust T cell responses as compared with those encountered during primary infection (14). However, these features of T cells have also been speculated to elevate the susceptibility of individuals to severe forms of dengue disease (15, 16).

In this context, the goal of our study was to assess the relationship between the magnitude of naturally acquired T cell memory resulting from one or more DENV infections and their impact on a subsequent DENV infection. This study aimed to expand our understanding of the role of preexisting DENV-specific T cells in either providing protective immunity or triggering immunopathogenic responses leading to clinical disease. We hypothesized that a higher preexisting frequency of CD4^+^ and CD8^+^ T cells would be associated with protection from subsequent symptomatic DENV infection. We analyzed T cell responses using flow cytometry, evaluating the phenotypes and frequencies of CD4^+^ and CD8^+^ T cells individually and examining relationships with the outcome of subsequent DENV infection. Importantly, we were able to test our question using a substantial sample size (*n* = 71), together with accurate characterization of infection history, information about the HLA type of the donors, and our established DENV peptide MegaPool (MP) approach to asses DENV-specific T cell responses (17). We hereby provide a comprehensive assessment of T cell memory in multiple DENV infections in an endemic setting.

## Results

### Pediatric cohort characteristics.

Our study used preinfection samples obtained from 71 children enrolled in the Pediatric Dengue Cohort Study, which has been ongoing in Managua, Nicaragua, since 2004. These samples were collected between 2006 and 2019 and grouped based on the outcome of the subsequent DENV infection: 46 participants who subsequently developed inapparent DENV infections (referred to herein as the preinapparent group) versus 25 participants who later experienced symptomatic DENV infection (referred to herein as the presymptomatic group) (Table 1). The mean age was 9 years in the preinapparent group and 11 years in the presymptomatic group. In the preinapparent infection group, 48% of participants were male and 52% were female. In the presymptomatic infection group, 64% were male and 36% were female. The age distribution was examined for balance between groups, yielding a *P* value of 0.0799. Additionally, the association between sex and the preinapparent or presymptomatic group was assessed, and the results indicated no statistically significant difference. In summary, the groups can be characterized as being well balanced. Furthermore, to categorize the participants, we considered their infection history, distinguishing between those who had encountered only 1 prior DENV infection (referred to as 1 DENV) and those who had experienced 2 or more previous DENV infections (referred to as 2+ DENV). Among the individuals in the preinapparent infection group (who remained protected against subsequent dengue symptoms), 61% had a history of 1 previous DENV infection and 39% had experienced 2 or more previous infections. Within the presymptomatic group, 44% had experienced 1 previous DENV infection, while 56% had experienced 2 or more DENV infections (Table 1).

### DENV-specific CD4^+^ T cell memory elicited by previous natural DENV infections.

DENV-specific CD4^+^ T cell memory responses were evaluated following a 24-hour stimulation of PBMCs with DENV CD4 MPs and using an activation-induced marker (AIM) assay as readout. Our laboratory developed the MP approach to study the breadth of T cell responses to diverse pathogens (17). In previous studies, HLA class I– and HLA class II–restricted DENV epitopes spanning the entire sequence of the DENV genome were predicted and subsequently experimentally tested in different human cohorts from endemic regions (10, 18, 19). In this study, we used 2 different DENV-specific MPs, 1 tailored to measure CD4^+^ T cell responses (containing 180 epitopes) and 1 tailored to measure CD8^+^ T cell responses (containing 268 epitopes).

Consequently, the CD4^+^ T cell reactivity to DENV MPs assessed by the AIM assay is represented as upregulation of the markers OX40^+^CD137^+^ on CD4^+^ T cells following activation (20–22). T cell responses were calculated by subtracting individual CD4^+^ T cell responses to the negative control (PBMCs only incubated with the DMSO solvent for 24 hours) from each individual’s CD4^+^ T cell response to DENV MP. Upon direct comparison of the geometric mean of the AIM^+^CD4^+^ T cells between the preinapparent and presymptomatic group, no statistically significant differences could be detected.

**Figure 1:**
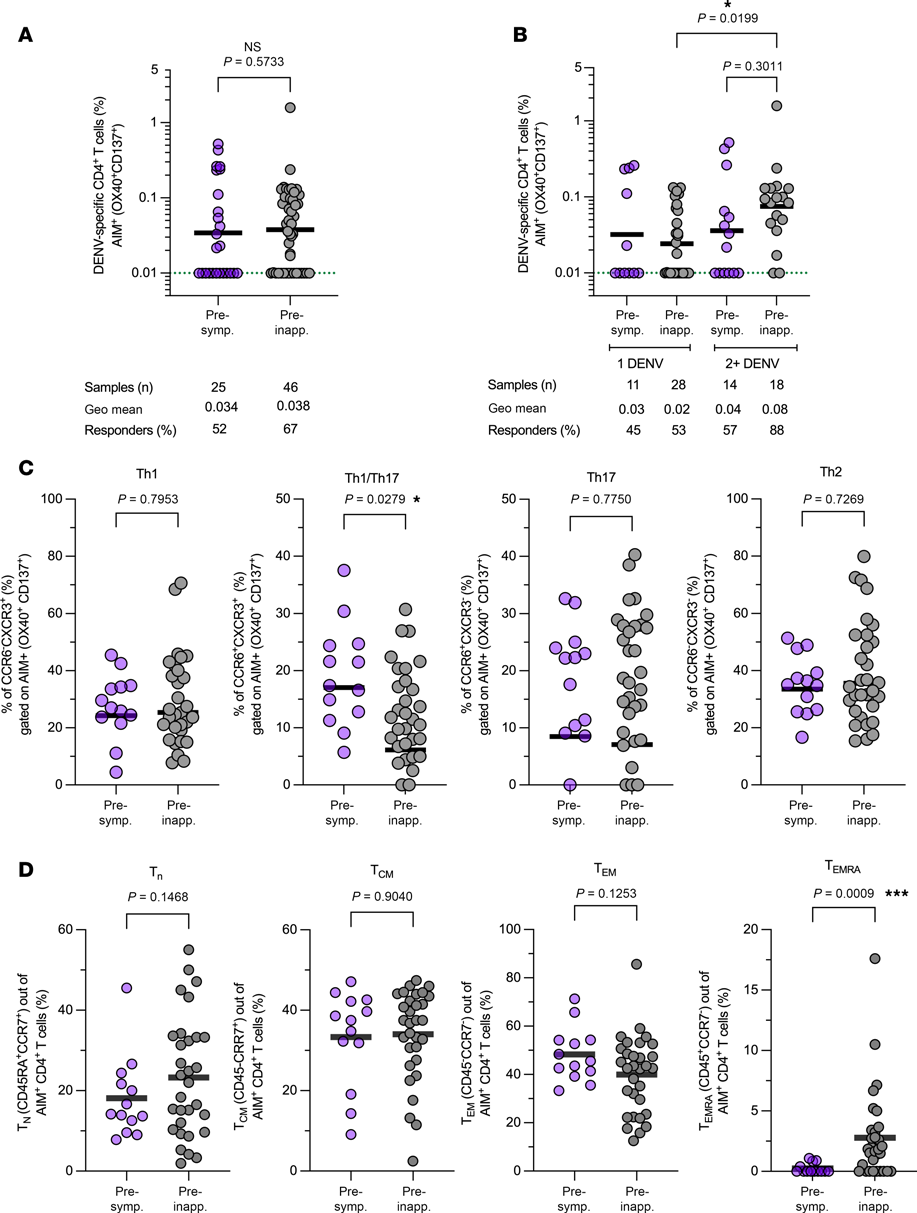
DENV-specific CD4^+^ T cell memory elicited by previous natural infections. (**A**) DENV-specific CD4^+^ T cell memory responses between preinapparent (gray circles) and presymptomatic (purple circles) groups were compared. (**B**) DENV-specific CD4^+^ T cell responses were stratified based on the number of prior DENV infections. The dotted green line indicates the limit of quantification (LOQ). Baseline and nonresponders were set at 0.01 of LOQ. Bars represent the geometric mean. Data were analyzed for statistical significance using the Mann-Whitney test. (**C**) Th cell subset frequency within AIM^+^CD4^+^ T cells was analyzed based on chemokine receptor expression using CXCR3 and CCR6 as markers. Bars represent the geometric mean. (**D**) Distribution of memory T cell subsets was defined within AIM^+^CD4^+^ T cells based on CCR7 and CD45RA expression as T cell naive (Tn), T central memory (Tcm), T effector memory (Tem), and T effector memory reexpressing CD45RA (Temra) cells. Bars represent the geometric mean. For ancestral gating and representative flow cytometry plots of DENV-specific CD4^+^ T cells (OX40^+^CD137^+^), see Supplemental Figure 2.

In this study, the term responder is used to describe T cells that exhibit a measurable upregulation of activation markers in response to the DENV MP stimulation. Within the presymptomatic group, there was a lower frequency of responders (52%) compared with that in the preinapparent group (67%) (Figure 1A). Next, we stratified groups based on the number of prior DENV infections they had experienced in the past. The frequency of responders was higher in both groups after 2+ DENV infections compared with the frequency of responders after only 1 DENV infection (presymptomatic, 45% vs. 57%; preinapparent, 53% vs. 88%). There was no significant difference between the presymptomatic and preinapparent groups associated with the outcome of subsequent infections following a single DENV exposure. While we observed higher frequencies of DENV-specific CD4^+^ T cells in the preinapparent group compared with the presymptomatic group following 2+ DENV infections, this difference was not statistically significant (*P* = 0.3011). When examining the magnitude of AIM^+^ responses within the preinapparent group, a significant difference emerged between those with 1 DENV prior infection and those with 2+ DENV prior infections, suggesting that more than 1 DENV infection may be necessary to induce a robust CD4^+^ T cell memory response (Figure 1B). Thus, we do not claim that the accumulation of memory CD4^+^ T cells alone predicts protection. The key observation is that memory CD4^+^ T cells tend to accumulate in correlation with each prior infection in the preinapparent group. Our results suggest a trend toward higher frequency of memory CD4^+^ T cells in preinapparent cases with 2+ DENV infections, although this trend did not reach statistical significance.

Subsequently, we assessed PD-1 expression within AIM^+^CD4^+^ T cells based on the history of infections. The frequency of PD-1 expression among AIM^+^CD4^+^ T cells did not show an accumulation or increase in children with a higher number of dengue cases or a significant difference between the preinapparent and presymptomatic groups (Supplemental Figure 6A; supplemental material available online with this article; https://doi.org/10.1172/jci.insight.179771DS1).

We further analyzed the phenotype of DENV-specific CD4^+^ T cells (AIM^+^) using antibodies against chemokine receptors with preferential expression on functionally distinct memory T cell subsets. This analysis describes 4 distinct Th cell subsets based on chemokine receptor expression: CXCR3^+^CCR6^−^ and CXCR3^+^CCR6^+^ (both enriched in Th1/Th17 cells) as well as CXCR3^–^CCR6^+^ (enriched in Th17 cells) and CXCR3^–^CCR6^–^ (enriched in Th2 cells). The preinapparent group demonstrated significantly higher levels of the DENV-specific memory T cells characterized by CXCR3^+^CCR6^+^ expression as compared with the presymptomatic group (Figure 1C). We have included an analysis of participant infection history in Supplemental Figure 3A. This additional analysis shows no significant differences between the groups.

Next, we enumerated the DENV-specific CD4^+^ T cell memory subsets by employing a combination of CD45RA and CCR7 markers. This approach has been published as a gold standard for identifying memory T cell subsets (23–26). We found that, in all analyzed participants regardless of the stratification by infection history, the DENV-specific CD4^+^ T cells were predominantly composed of effector memory T (Tem) cells (CD45RA^−^CCR7^−^), followed by central memory T (Tcm) cells (CD45RA^−^CCR7^+^), and then the T effector memory reexpressing CD45RA (Temra) subset (CCR7^+^CD45RA^+^), which was the smallest subset. Regarding the distribution of these memory T cell subsets, the geometric mean frequency varied. For Tem cells, the mean frequency was 47% in the presymptomatic group compared with 37% in the preinapparent group. Tcm cells exhibited a frequency of 30% in the presymptomatic group and 31% in the preinapparent group, with no significant differences observed between the two groups. However, the Temra subset (CCR7^+^CD45RA^+^) was significantly different between the preinapparent and presymptomatic groups (*P* = 0.009), constituting a very low percentage (0.25%) in the presymptomatic group compared with a significantly higher percentage (2.8%, *P* = 0.0009) in the preinapparent group (Figure 1D). We have included an analysis that considers the infection history in Supplemental Figure 3B. This additional analysis revealed a difference with respect to infection outcome, within the Temra population following 2 or more prior DENV infections. Furthermore, we analyzed the frequency of DENV-specific circulating T follicular helper (cTfh) cells, defined as CXCR5^+^OX40^+^CD40L^+^, in the studied groups. Consistent with expectations, we found very low frequencies of these cells, which is likely due to their low presence in blood (Supplemental Figure 4).

### DENV-specific CD8^+^ T cell memory elicited by previous natural DENV infections.

**Figure 2:**
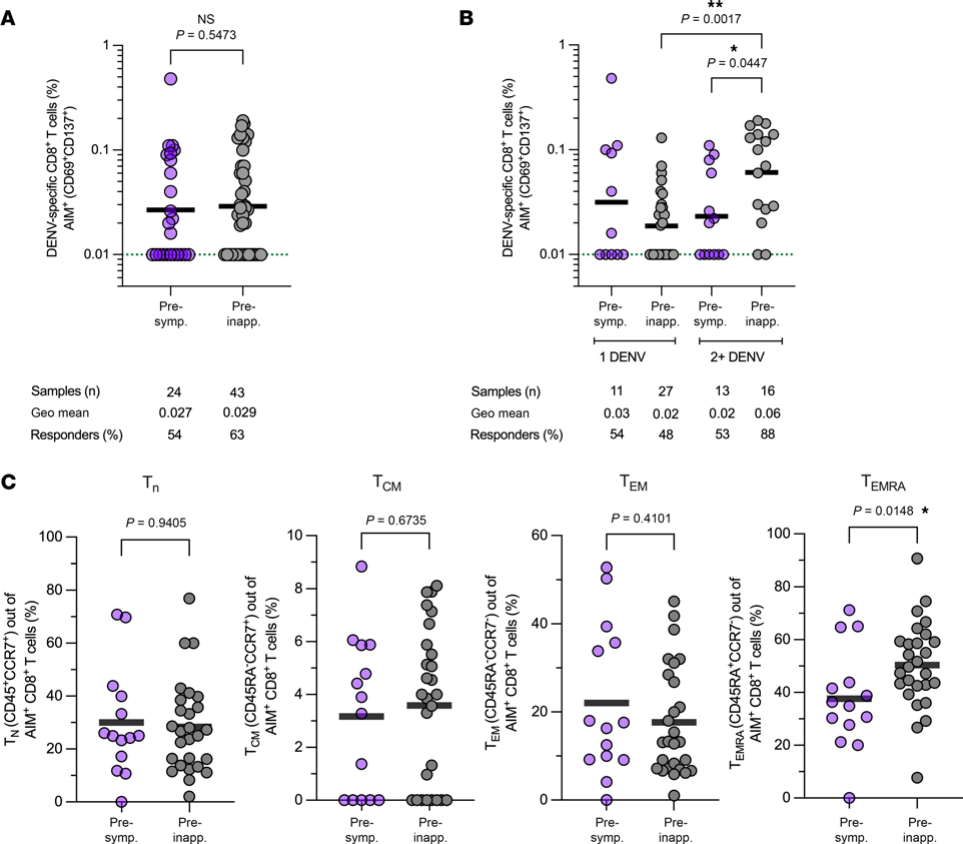
DENV-specific CD8^+^ T cell memory elicited by previous natural infections. DENV-specific CD8^+^ T cell memory responses were assessed with the AIM assay following a 24-hour stimulation of PBMCs with DENV CD8 MPs. T cell responses were calculated as background-subtracted responses to DENV CD8 MPs per individual. The dotted green line indicates the limit of quantification (LOQ). Baseline and nonresponders are set at 0.01 of LOQ. Bars represent geometric mean. Data were analyzed for statistical significance using the Mann-Whitney test. (**A**) DENV-specific CD8^+^ T cell memory responses between preinapparent and presymptomatic groups were compared. (**B**) DENV-specific CD8^+^ T cell responses were stratified based on the number of prior DENV infections. (**C**) The distribution of memory T cell subsets was defined within AIM^+^CD4^+^ T cells based on CCR7 and CD45RA expression. For ancestral gating and representative flow cytometry plots of DENV-specific CD8^+^ T cells (CD69^+^CD137^+^), see Supplemental Figure 2.

Analysis of the memory DENV-specific CD8^+^ T cell response was performed after a 24-hour stimulation with DENV-specific CD8 MP and using the AIM assay as readout. After activation, the reactivity of CD8^+^ T cells to DENV MPs, evaluated through the AIM assay, was indicated by an increase in the coexpression of CD69^+^ and CD137^+^ markers on CD8^+^ T cells. Upon direct comparison of the preinapparent and presymptomatic groups, there were no statistically significant differences between the geometric mean of the AIM^+^ responses: 54% of responders were observed in the presymptomatic group and 63% of responders were observed in the preinapparent group (Figure 2A).

Subsequently, upon stratification of these groups based on the participants’ history of prior DENV infections, differences in the magnitude of the AIM^+^ response once again became apparent. A noteworthy increase in AIM^+^ response within the preinapparent group was observed following 2+ DENV infections as compared with the presymptomatic group (*P* = 0.0447). In addition, in the preinapparent group, the frequency of CD8^+^ T cell memory responders was significantly higher after 2+ DENV infections (88%) as compared with only 1 DENV infection (48%) (Figure 2B). When comparing the magnitude of the AIM^+^ response exclusively within the preinapparent group, analogous to the observations made for CD4^+^ T cells, a significant difference between participants who had experienced only 1 DENV infection and those who had encountered 2+ DENV infections was observed (*P* = 0.0017). In conclusion, our results suggest that multiple DENV infections may be necessary to trigger a robust DENV-specific CD8^+^ T cell memory response (Figure 2B).

Finally, we investigated the memory subsets of DENV-specific CD8^+^ T cells by employing a combination of CD45RA and CCR7 markers. Irrespective of the participants’ infection history, DENV-specific CD8^+^ T cells were predominantly composed of the Temra subset (CCR7^+^CD45RA^+^). Specifically, this subset constituted 38% of the presymptomatic group compared with a higher percentage of 49% in the preinapparent group. Additionally, a significant difference was observed within the Temra subset (CCR7^+^CD45RA^+^) when comparing the 2 groups (*P* = 0.0148). No significant differences by infection history were observed among the percentages of naive, Tcm, and Tem cell subsets in terms of the geometric mean (Figure 2C). We have included an analysis that takes participant infection history into account in Supplemental Figure 5. The additional analysis showed no significant differences but exhibited a trend (*P* = 0.0608) toward enrichment of Temra cells after 2 or more prior DENV infections.

### Association of AIM^+^CD4^+^ T cell responses with preexisting DENV antibody titers.

**Figure 3:**
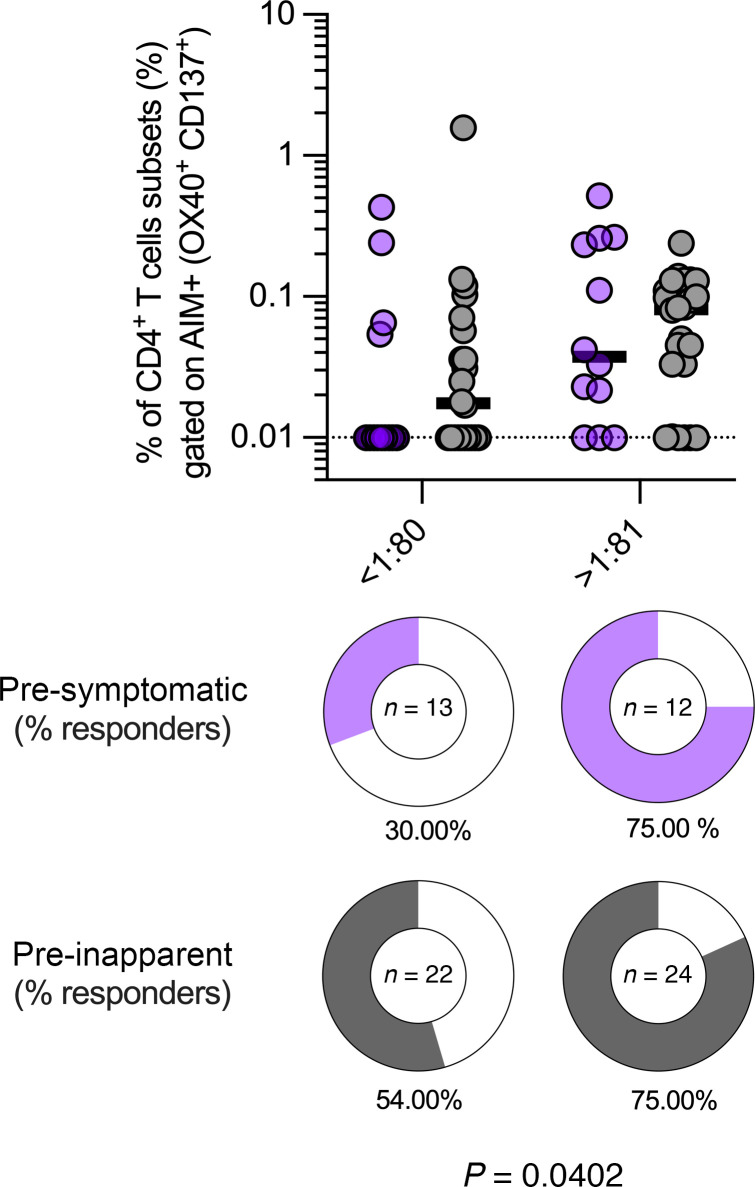
Association of AIM^+^CD4^+^ T cell responses and preexisting DENV antibody titers. AIM^+^CD4^+^ T cell magnitude and preexisting DENV antibody titer in presymptomatic and preinapparent groups were plotted side by side. AIM^+^ response frequencies are depicted in pie charts. While overall AIM^+^CD4^+^ T cell responses showed no significant differences (Kruskal-Wallis test) when compared by antibody titer level, statistically significant differences were found when the frequency of responders was compared within the antibody titer bins and between groups using the Fisher’s exact test.

CD4^+^ T cells promote B cell stimulation and consequently B cell–derived antibody responses; thus, we sought to delineate the relationship between preexisting CD4^+^ T cells and DENV binding antibody titer. We examined the association of the magnitude of AIM^+^CD4^+^ T cells in presymptomatic and preinapparent groups with their corresponding preexisting DENV antibody titers. When comparing the overall magnitude of DENV-specific AIM^+^CD4^+^ T cell responses within bins of DENV inhibition ELISA (iELISA) antibody titer, we did not note any statistically significant differences. However, differences emerged when we compared the frequencies of responders in each group. These are visually represented in Figure 3 using pie charts. In the context of an antibody titer lower than <1:80, only 30% of individuals in the presymptomatic group exhibited AIM^+^ responses, while this proportion was notably higher, at 54%, in the preinapparent group. Considering responders with antibody titer ranges of higher than >1:80, AIM^+^ responses were observed in 75% of presymptomatic individuals, a finding that aligns with the 75% of individuals in the preinapparent group with AIM^+^ responses. Importantly, the differences in response frequencies were determined to be statistically significant (*P* < 0.0402) through Fisher’s exact test (Figure 3). In conclusion, we found a significant association between the proportion of preexisting AIM^+^CD4^+^ T cell memory responders and DENV iELISA titer bins that was more pronounced in the preinapparent group than the presymptomatic group.

### HLA associations with disease outcome of the subsequent infection.

Polymorphisms within HLA alleles, specifically classical HLA-A and HLA-B class I alleles, have been linked to various outcomes in DENV infections, including resistance, susceptibility, and disease severity (27). Thus, we explored the effect of HLA alleles on the outcome of subsequent DENV infection. Table 2 summarizes results obtained through the application of the RATE tool from the Immune Epitope Database (http://iedb-rate.liai.org). RATE, which stands for Restrictor Analysis Tool for Epitopes, is an automated method designed to infer HLA restriction for a set of given epitopes from large datasets of T cell responses in HLA-typed individuals. The input for this analysis comprised HLA-type information from our donors and the outcome of the subsequent infection. Table 2 shows the allele number and corresponding allele name as denoted in the input data. The column labeled “A+ symptomatic+” represents the number of individuals expressing the allele and subsequently experiencing symptomatic infections. The “A+ symptomatic+ %” column provides the frequency of individuals for each outcome-allele combination within the cohort. Additionally, the “*P* value” column displays the *P* values obtained from Fisher’s exact test, offering a relative ranking that indicates which HLA restriction has the strongest association with symptomatic infection outcome. Our findings revealed 3 notable associations with subsequent symptomatic outcomes. One of these associations involved HLA-B*51:01, which had previously been associated with the development of dengue hemorrhagic fever in patients experiencing secondary infections (28, 29). Additionally, we also found associations with HLA-A*02:05 and HLA-B*15:10, which to our knowledge had not been previously described in the context of DENV infections. Collectively, our results deliver genetic evidence that underscores the potential role of classical HLA class I alleles in shaping the outcome of subsequent DENV infection (Table 2 and Supplemental Table 2). Table 2 presents associations between HLA-A and HLA-B class I alleles and subsequent DENV infection outcomes. The complete association matrix tables can be found in Supplemental Table 2.

## Discussion

In our study of a long-term pediatric cohort in Managua, Nicaragua, we examined memory T cell responses to naturally acquired DENV infections. We focused on 71 healthy children with 1 or more prior DENV exposures before a subsequent DENV infection within the following year. Our primary goal was to investigate how preexisting DENV-specific T cell responses affected the outcome of subsequent dengue infection as inapparent or symptomatic. To investigate the presence of DENV-specific T cells in these healthy children, we employed the AIM assay. This approach, which is based on T cell receptor–dependent activation marker upregulation, allowed us to capture a diverse and cytokine-independent T cell response (22).

Utilizing this method, we detected DENV-specific CD4^+^ T cells in 61% and DENV-specific CD8^+^ T cells in 59% of all children with prior DENV infection. When considering the entire cohort, we did not identify discernible differences in the levels of overall DENV-specific CD4^+^ and CD8^+^ T cell responses between children who subsequently developed symptomatic infections and those who developed inapparent infections. However, when groups were stratified by infection history, DENV-specific CD4^+^ and CD8^+^ T cell responses exhibited a higher magnitude after 2 infections only in children with subsequent inapparent infections. Furthermore, DENV-specific CD8^+^ T cell responses exhibited a higher magnitude after 2 infections in children with subsequent inapparent infections compared with children with subsequent symptomatic infections. This phenomenon in children with a history of more than 2 prior DENV infections suggests a potential requirement for multiple DENV infections to induce a robust T cell memory response.

The definitive role of preexisting DENV-specific T cells has not been unanimously defined. While higher preexisting T cell responses have previously been associated with disease (19, 30, 31), our findings diverge, as we found that a higher frequency of preexisting DENV-specific T cells was associated with the subsequent inapparent outcome of infection. Notably, the aforementioned study employed a cultured in vitro ELISPOT assay that did not distinguish between the relative contributions of different T cell subsets. Additionally, the study cohort was relatively small, and the methodology involved the use of peptides with a length suitable to favor CD4^+^ T cell responses (32). In contrast, in a DENV human challenge model, IFN-γ from T cells in acute DENV infection was associated with a protective role in vivo (33). In agreement, our study showed a significant association between the magnitude of DENV-specific T cells and a subsequent inapparent DENV infection outcome.

Additionally, the results from our study revealed a significant Th1/Th17 polarization within DENV-specific CD4^+^ T cells in children who experienced subsequent symptomatic infections. This subset has previously been described within the memory CD4^+^ T cells from patient cohorts experiencing a latent *Mycobacterium tuberculosis* infection (34), but to the best of our knowledge, it is the first description in the context of DENV T cell memory. In PBMCs from patients experiencing dengue hemorrhagic fever, flow cytometry analysis after nonspecific polyclonal stimulation demonstrated a strong immune response skewed toward IL-17 production, marked by a heightened occurrence of CD4^+^IL-17^+^ T cells (35). This suggests that Th17-polarized cells could contribute to symptomatic or severe disease in DENV infection. Additionally, chemokine receptors could play an important role in mediating T cell recruitment to the site of inflammations. The expression of CCR6 and CXCR3 on T cells reflects their diverse capacities for trafficking to nonlymphoid tissues (23). The source and differentiation requirements of this particular Th1/Th17 subgroup, along with their potential functions, require further clarification. Future studies should investigate whether this Th1/Th17 subset synergistically collaborates with other proinflammatory cytokines, leading to an augmentation of systemic inflammation. When stratifying the samples by the number of prior DENV infections (either 1 or 2+), we observed that the Th1/Th17 polarization differences in DENV-specific CD4^+^ T cells were no longer apparent, indicating that repeat infection is necessary to induce this Th1/Th17 subset.

When analyzing the memory cell subsets within the AIM^+^ DENV-specific T cells, we found a higher frequency of Temra cells in CD4^+^ T cells and CD8^+^ T cells among children with subsequent inapparent infections. Temra cells have been described as a subset of CD4^+^ T cells known for their cytotoxic abilities, as indicated by surface exposure of CD107a, a molecule that is translocated to the outside of the cell membrane upon degranulation and the release of cytotoxic molecules such as granzyme B and perforin (36). Temra cells have been described by us and others in individuals with both primary and secondary DENV infections, with varying numbers based on infection history. Temra CD4^+^ and CD8^+^ T cells have been detected in secondary DENV infections, indicating that repeated exposure to DENV antigens triggers their development (30, 37). Notably, DENV-specific Temra cells are more abundant in patients with mild dengue fever compared with severe cases, implying a protective role (16). In summary, our results demonstrate for the first time to our knowledge that preexisting CD4^+^ and CD8^+^ Temra cells are significantly associated with subsequent inapparent infection outcome, particularly in those with a history of repeated exposure.

In our analysis of cTfh cells, defined as CXCR5^+^OX40^+^CD40L^+^ cells, we observed a notably low frequency in children whose DENV infections occurred more than 3 years ago. This aligns with established findings that DENV-specific cTfh cells in peripheral blood are scarce after acute infection. After differentiation, Tfh cells predominantly reside in secondary lymphoid organs, like lymph nodes and the spleen, where they play a critical role in supporting B cells. In contrast, other effector CD4^+^ T cell subsets typically leave these lymphoid tissues to migrate toward sites of infection or inflammation (38). Additionally, while peripheral blood contains memory CXCR5^+^CD4^+^ T cells — often referred to as blood Tfh cells — which share functional and phenotypic similarities with Tfh cells, these cells are generally more abundant in specific contexts, such as following vaccination, during autoimmune responses, or in chronic viral infections. In combination with other markers like CCR7 and PD-1, which are associated with the memory phenotype, these cells can persist in circulation (39–41). However, the low frequency of memory cTfh cells in the studied cohort, particularly more than 3 years after DENV infection, likely reflects the long-term residency of Tfh cells within lymphoid tissues. This low prevalence in peripheral blood underscores the dynamic redistribution of cTfh cells, which are retained in lymphoid organs to fulfill their primary function of B cell assistance, rather than circulating in significant numbers over time.

We observed that PD-1 expression was significantly elevated in AIM^+^CD4^+^ and CD8^+^ T cells during acute DENV infection, as shown in Supplemental Figure 5. This supports the notion that PD-1 may act as an activation marker rather than an indicator of exhaustion in this context. The role of PD-1 as a marker for T cell function is context dependent, serving as a marker of activation, exhaustion, or suppression depending on the circumstances (37, 42, 43). To accurately characterize PD-1–expressing T cell subsets, additional markers are required. Specifically, PD-1 expression in AIM^+^CD4^+^ and CD8^+^ T cells must be carefully evaluated. This expression suggests that PD-1 may not necessarily indicate T cell exhaustion, particularly in the context of an acute DENV infection, as opposed to a chronic, antigen-persistent condition (44). Furthermore, while PD-1 is commonly associated with exhaustion, it is also recognized as an activation marker in acute infections, potentially being upregulated following peptide restimulation and T cell expansion.

In our study, we describe a significant relationship between HLA types and the subsequent outcome of DENV infections. Our findings not only corroborate the previously reported association involving HLA-B51:01 but also introduce two additional HLA types linked to subsequent symptomatic infections. Understanding HLA disease associations is crucial, as it sheds light on the genetic factors that influence susceptibility to specific diseases.

In summary, we detected DENV-specific CD4^+^ T cells and DENV-specific CD8^+^ T cells in approximately 60% of children in our study. Notably, children with 2+ prior DENV infections had significantly higher levels of DENV-specific CD8^+^ T cells than those with only 1 prior DENV infection. Furthermore, children with a subsequent symptomatic infection exhibited significant Th1/Th17 polarization in DENV-specific CD4^+^ T cells. Additionally, children with inapparent infections had a higher frequency of preexisting Temra T cells in DENV-specific CD4^+^ and CD8^+^ T cells. The relationship between higher DENV-specific memory T cells and subsequent inapparent DENV infections in children after 2+DENV infections, highlights a protective role for these cells. Importantly, the protective role of preexisting T cell memory was enhanced in children with multiple prior infections. This is important to consider in the context of DENV vaccination programs, where a number of previous natural infection may occur in endemic settings.

Our study demonstrates the ability to detect antigen-specific T cells in healthy children with a history of DENV infections by analyzing the coexpression patterns of AIMs on T cells. Experiencing 2 or more DENV infections was associated with the presence of DENV-specific T cells. Importantly, heightened DENV-specific T cell presence was correlated with an absence of clinical symptoms during subsequent DENV infections, suggesting a protective role for DENV-specific T cells in DENV infections.

### Limitations of our study.

The study we conducted has several limitations that need to be taken into account when interpreting our findings. The time elapsed since the last DENV infection in our study participants ranged from approximately 3–5 years. T cell responses can be highly sensitive to temporal factors and change over time. Our study examined a single time point before a subsequent DENV infection, with two possible outcomes. This means we were not able to describe the dynamic changes in the T cell response over time. Finally, our study focused on examining PBMCs within the bloodstream of healthy children. These cells may not provide an accurate reflection of the DENV-specific T cell repertoire developed in response to previous DENV infections, as this repertoire is predominantly localized to tissues like the skin, where the initial virus inoculation occurs. The variations in infection dynamics, initial viral load, and the localization of the T cell memory repertoire could potentially influence the outcome of subsequent DENV infections that cannot be assessed with our approach. Due to limited blood volume in pediatric samples, we focused on the AIM assay, which allowed us to assess virus-specific T cells in an antigen-specific, cytokine agnostic way, assessing all responding DENV-specific T cell subsets. The limited blood volume further restricted our ability to perform additional functional analyses of T cell subsets, which may have provided a more comprehensive understanding of T cell function. Finally, the small sample size restricted the power of our HLA association analyses, and we were unable to directly test the recognition of B5101-restricted peptides in the same donors owing to sample limitations. This should be considered when interpreting our findings.

## Methods

### Sex as a biological variable.

Both male and female participants were included across all groups; female participants made up 46.5% and male participants made up 53.5% of the study group. However, the study was not designed to assess sex-specific differences, as its primary focus was unrelated to sex as a variable.

### Study population.

A total of 103 peripheral blood samples were collected from participants enrolled in the Pediatric Dengue Cohort Study, an ongoing prospective cohort spanning 2 decades, centered on children aged 2–17, in Managua, Nicaragua. A description of this cohort has been outlined previously in ref. 45. To briefly elucidate, upon enrollment, parents committed to promptly seek medical attention for their children upon the emergence of initial symptoms. Validation of cases presenting symptomatic dengue was accomplished via the identification of DENV RNA using RT-PCR, real-time RT-PCR, and/or virus isolation within the acute-phase sample (within 0–6 days after symptom onset).

### Serology.

Evaluation of inapparent DENV infections was performed annually by comparing serum samples obtained from 2 consecutive healthy years utilizing iELISA as detailed in Katzelnick et al. (8). An iELISA titer increase exceeding 4-fold between yearly samples in children who did not seek medical care at the study health center in Managua, Nicaragua, between the annual samplings is considered an inapparent infection (46).

### Definition of history of infection for 1 DENV.

This group includes individuals who had experienced only 1 prior DENV infection during the observational period of the study. The classification as 1 DENV was determined by documentation of a single prior DENV infection based on molecular, virological, and serological analysis of dengue cases and iELISA titers in paired healthy annual samples.

### Definition of history of infection for 2+ DENV.

This group encompasses individuals who had encountered 2 or more prior DENV infections within the study period. These participants were classified as 2+ DENV based on documented evidence of multiple DENV infections, including laboratory-confirmed dengue cases and infections identified by iELISA titers in paired annual samples.

### Exclusion criteria.

In our study, we initially examined 103 samples, but upon unblinding, 32 donors were identified as “DENV immune.” Owing to the uncertainty about the amount of previous DENV infections and the timing of their last infection, we opted to exclude these donors from our analysis. This decision was made to preserve the accuracy of our research, as both the exact number of past DENV infections and the time frame of these infections are pivotal factors in our analysis of the influence of preexisting T cells in the outcome of subsequent DENV infections.

### PBMC isolation.

Human blood samples were subjected to density gradient centrifugation using Ficoll-Paque Premium (GE Healthcare Biosciences). Subsequently, the isolated PBMCs were resuspended in fetal bovine serum (Gemini Bio-products; Gibco Life Technologies) containing 10% dimethyl sulfoxide and cryopreserved in liquid nitrogen, following established protocols described in ref. 18.

### DENV MPs.

The MP approach was previously established by Sidney et al. (47) and recently detailed protocols were made public by R. da Silva Antunes et al. (17), allowing the simultaneous assessment of numerous T cells from diverse specificity. Briefly, this method consists of the dissolution of a diverse array of epitopes, their consolidation into a combined pool, and subsequent joint lyophilization to avoid cell cytotoxicity by a larger amount of DMSO. In this study, PBMCs were ex vivo stimulated to enable the examination of antigen-specific T cell responses against the DENV using flow cytometry. The employed DENV-specific peptide pool for CD8^+^ T cells was composed of 268 overlapping 9- and 10-mers, and the DENV-specific CD4^+^ T cell peptide pool was composed of 180 overlapping 15-mers as previously described by (19, 48).

### T cell assays.

All T cell assays were carried out by two independent investigators on the same spectral analyzer (Cytek Aurora, Cytek Biosciences). The samples were distributed in a blinded manner. Samples were run, including a positive (phytohemagglutinin) and negative control (DMSO), for each donor in the same experiment. Furthermore, each independent experiment included a sample of the same control donor, with known CD4^+^ and CD8^+^ T cell reactivity. Details of the T cell assays are provided below. After all experiments were run, experimentalists were unblinded to allow for specific analysis and data interpretation.

### AIM assay.

The AIM assays were performed following established methods described previously by ref. 21. An overview of the experimental design is provided in Supplemental Figure 1. The quantification of dengue-specific T cells was determined by assessing the proportion of AIM^+^ T cells, defined as OX40^+^CD137^+^ for CD4^+^ T cells and CD69^+^CD137^+^ for CD8^+^ T cells after a 24-hour PBMC stimulation. Briefly, before adding the dengue MP, PBMCs were subjected to a 15-minute blocking step at 37°C using 0.5 μg/mL anti-CD40 monoclonal antibody (Miltenyi Biotec). Following this, cells were incubated with fluorescently labeled chemokine receptor antibodies (anti-CCR6, CXCR5, CXCR3, and CCR7) and the dengue MPs (1 μg/mL) in 96-well U-bottom plates and incubated at 37°C, 5 % CO_2_, for 24 hours. Additionally, PBMCs were incubated with an equimolar quantity of DMSO for negative control purposes and with phytohemagglutinin (0.5 μg/mL) (Roche) as a positive control. For surface staining, 1 × 10^6^ PBMCs were resuspended in PBS, treated with human FC block from BD Bioscience, and stained with LIVE/DEAD Blue marker (Thermo Fisher Scientific) in darkness for 15 minutes, and then rinsed with PBS. Subsequently, the remaining surface antibodies were added to the cells and incubated for 60 minutes at 4°C in the dark. After surface staining, the cells were washed twice with PBS containing 3% FBS (FACS buffer). The cells were immediately acquired using a Cytek Aurora flow cytometer (Cytek Biosciences). The antibodies employed in this panel are listed in Supplemental Table 1, and the representative gating strategy for dengue-specific CD4^+^ and CD8^+^ T cells using the AIM assay is shown in Supplemental Figure 2.

The quantification of DENV-specific CD4^+^ and CD8^+^ T cells was achieved by subtracting the frequency of AIM^+^ cells in the unstimulated condition from the frequency in the antigen-stimulated setting. The minimum DMSO level was fixed at 0.005%. The fold change was calculated as the ratio of the frequency of AIM^+^ cells during antigen stimulation to that in the unstimulated state. Responses above 0.01% and a stimulation index greater than 1.5 were considered positive. In this study, we employed a comprehensive panel of markers, including CD45RA and CCR7, to identify and discriminate among various T cell subsets: CD45RA^+^CCR7^+^ naive (Tn), CD45RA^−^CCR7^+^ central memory (Tcm), CD45RA^−^CCR7^−^ effector memory (Tem), and CD45RA^+^CCR7^−^ effector memory reexpressing CD45RA (Temra) T cells (23–25, 49).

### HLA typing.

HLA typing for class I (HLA A, B, C) and class II (DRB1, DRB3/4/5, DQA1/DQB1, DPB1) was carried out by a laboratory accredited by American Society for Histocompatibility and Immunogenetics at Murdoch University in Western Australia, following established protocols as detailed previously, using locus-specific PCR amplification of genomic DNA. Table 1 lists the HLA types of all donors used in this study.

### RATE analysis.

Table 2 in the Supporting Data Values file presents associations between HLA-A and HLA-B class I alleles and subsequent DENV infection outcomes. The complete association matrix tables can be found in Supplemental Table 2. The association matrix was generated using the RATE tool (http://iedb-rate.liai.org).

The RATE tool was used to compute the HLA restrictions by considering the presence or absence of a given stimulus as the biological outcome. We uploaded the HLA-typing information and response data as.txt files. The cohort was divided into preinapparent and presymptomatic; the individuals classified as symptomatic had positive responses (value = 1), while the individuals classified as inapparent had a negative response (value = 0). The cutoff was established at 1 for the positive response because the response data was provided in binary values. The script calculated the participants’ relative frequency (RF) to a given stimulus and the expression of a given allele compared with the general test population and associated statistical significance. RF >1 indicates a positive association between the two properties in question, meaning that the expression of the specific allele increases the possibility of having a positive immune response. The RATE tool used Fisher’s exact test to estimate the statistical significance of the association between HLA molecules and stimuli responses. The RATE tool is a Python 2.6.5+ CGI script previously described and validated in ref. 50.

### Statistics.

Flow cytometry data analysis was conducted using FlowJo version 10.8.2. Statistical analyses were carried out using GraphPad Prism version 9.3.0 unless specified otherwise. Data presented in linear scales are represented as geometric mean. Unpaired comparisons were evaluated using Mann-Whitney. For multiple comparisons, Kruskal-Wallis tests followed by Dunn’s post test was employed. Additional details regarding significance are included in the respective figure legends. *P* values of less than 0.05 were considered significant.

### Study approval.

Samples were collected under the ethical approval provided by the Institutional Review Boards of the La Jolla Institute for Immunology (LJI VD-085); the University of California, Berkeley; and the Nicaraguan Ministry of Health.

### Data availability.

All the data generated in this study are available in the published paper and summarized in the corresponding tables, figures, and supplemental materials. Values for all data points in graphs are reported in the Supporting Data Values file. Any additional information required to reanalyze the data reported in this paper is available upon request. Requests should be directed to DW. Upon specific request and execution of a material transfer agreement, aliquots of the peptide pools utilized in this study can be made available. Limitations might be applied to the availability of peptide reagents due to cost, quantity, demand, and availability.

## Author contributions

Conceptualization was provided by DW and EH. Methodology was provided by RIG and EAE. Formal analysis was provided by RIG, EAE, and AMP. Investigation was provided by RIG, EAE, AMP, TS, JVZ, AB, EH, and DW. The project was administered by EH and DW. Funding was acquired by EH and DW. The manuscript was written by RIG, EH, and DW. Supervision was provided by AB, EH, and DW. All authors provided critical feedback and helped shape the research, analysis, and manuscript.

## Supplementary Material

Supplemental data

Supporting data values

## Figures and Tables

**Table 1:**
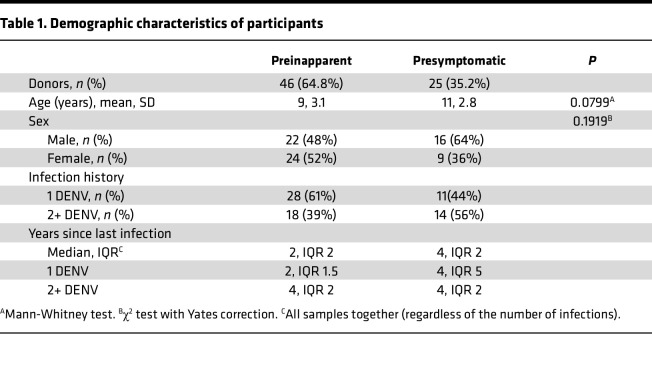
Demographic characteristics of participants

**Table 2:**
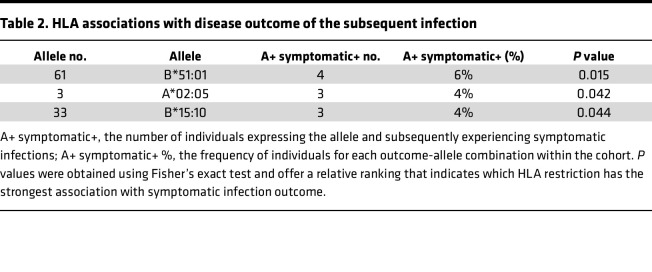
HLA associations with disease outcome of the subsequent infection

## Version 1 02/24/2025

Electronic publication
