# Supplementary material for: Frequency of dengue virus–specific T cells is related to infection outcome in endemic settings
Source: JCI Insight. 2025 Feb 24;10(4):e179771. doi: 10.1172/jci.insight.179771 (PMC11949061; doi:10.1172/jci.insight.179771)
Supplement: Supplemental data [file jciinsight-10-179771-s169.pdf]

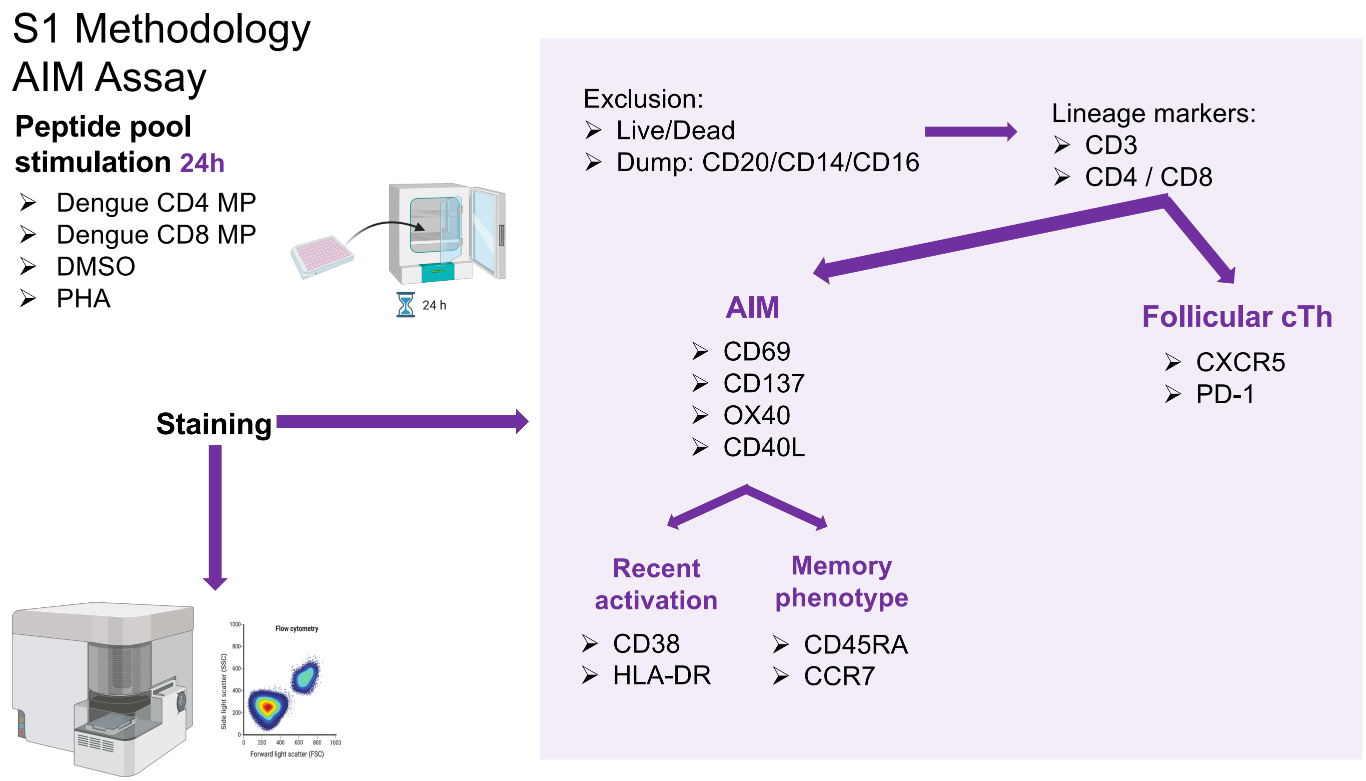

Figure S1. Representative experimental design for AIM Assay for T cell analysis.

S2 Gating Strategy

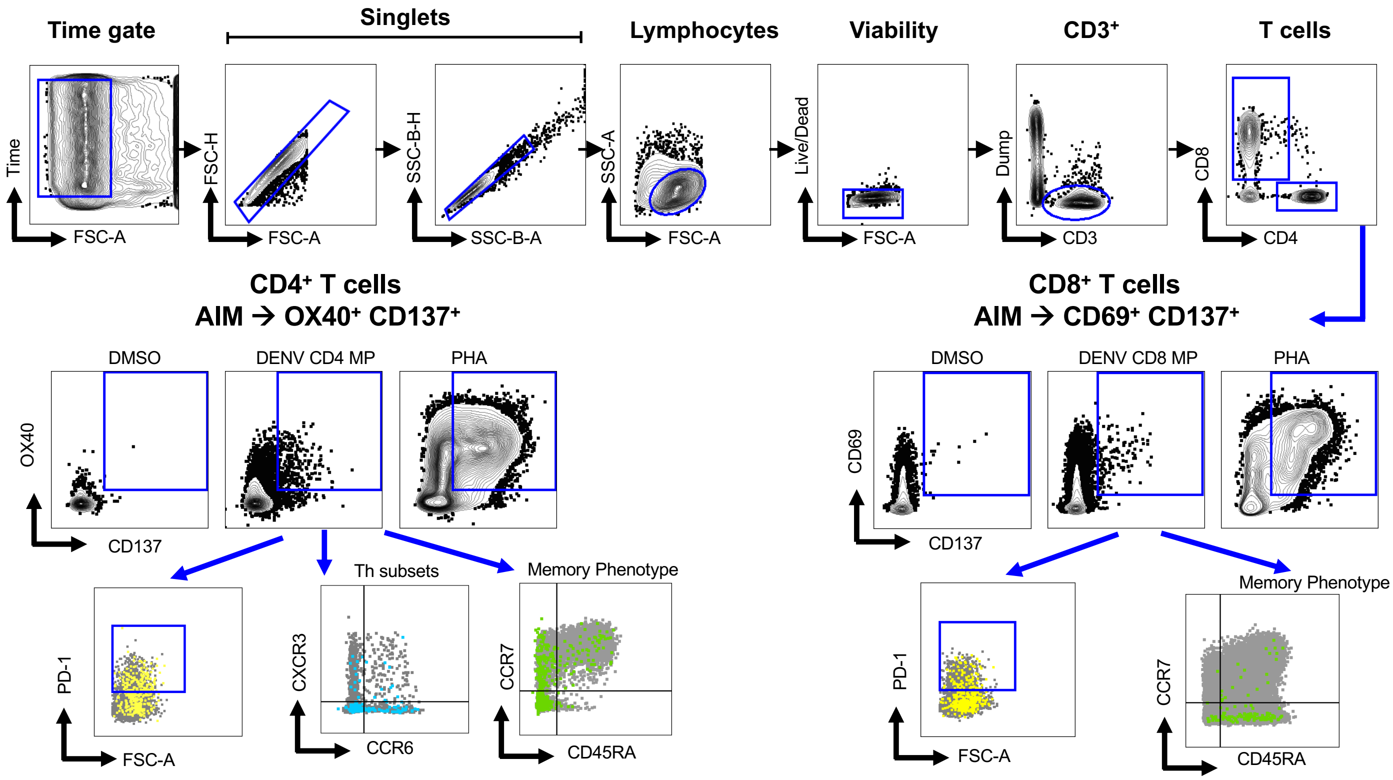

Figure S2. Representative gating strategy for DENV-specific T cell analysis

Related to Figures 2–3

(A) Representative strategy to define AIM$^+$ CD4$^+$ T cells: CD3$^+$CD4$^+$OX40$^+$CD137$^+$.

(B) Representative strategy to define AIM$^+$ CD8$^+$ T cells: CD3$^+$CD8$^+$CD69$^+$CD137$^+$.

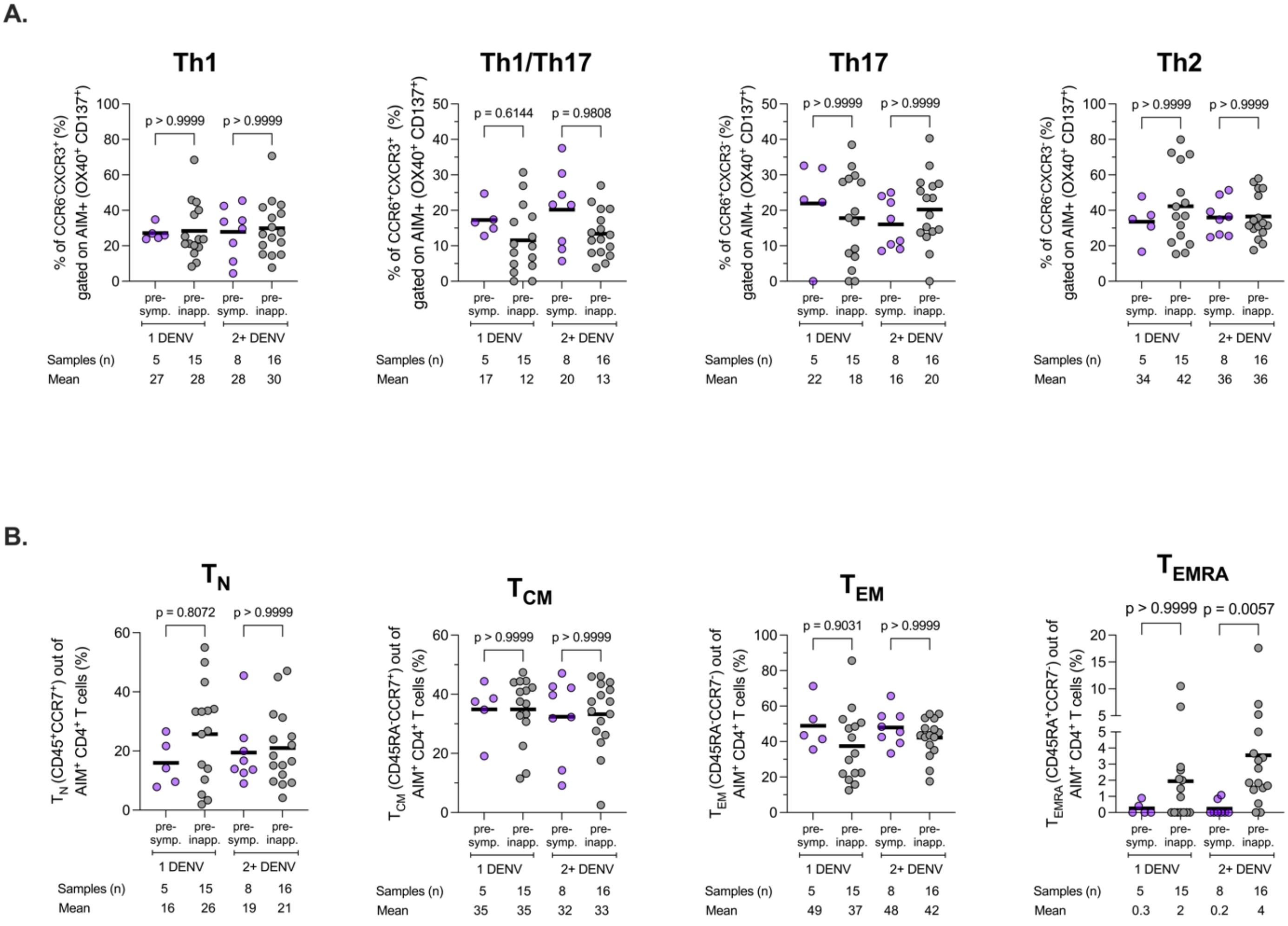

Figure S3. DENV-specific CD4⁺ T cell populations were stratified based on the number of prior DENV infections.

A. Four distinct T helper cell subsets (Th) based on chemokine receptor expression.

B. DENV-specific CD4+T cell memory subsets defined by CD45RA and CCR7.

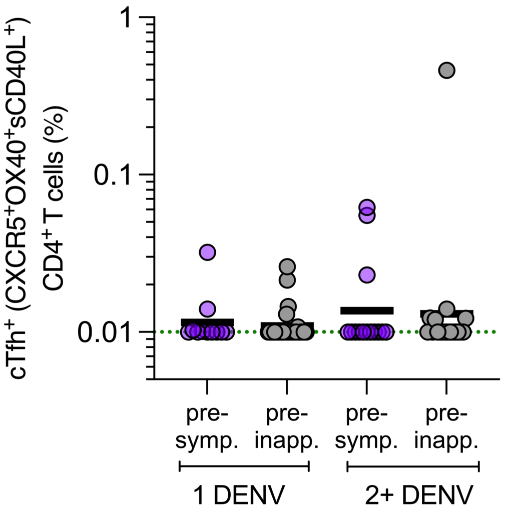

Samples (n)	11	28	14	18
Geo Mean	0.01	0.01	0.01	0.01
Responders (%)	36	18	21	28

Figure S4. DENV-specific cTfh cells stratified based on the number of prior DENV infections.

A.

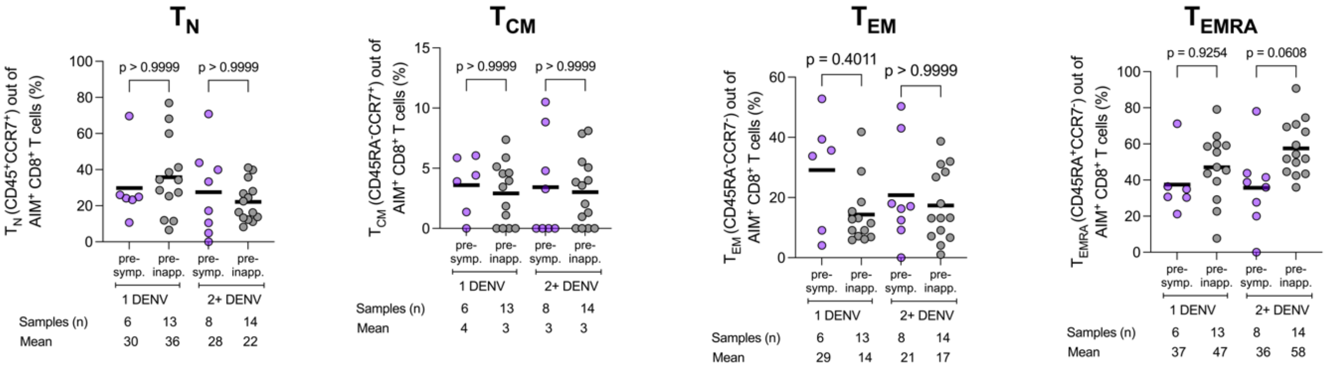

Figure S5. DENV-specific CD8^{+} T cell populations were stratified based on the number of prior DENV infections.

A. DENV-specific CD8+T cell memory subsets defined by CD45RA and CCR7.

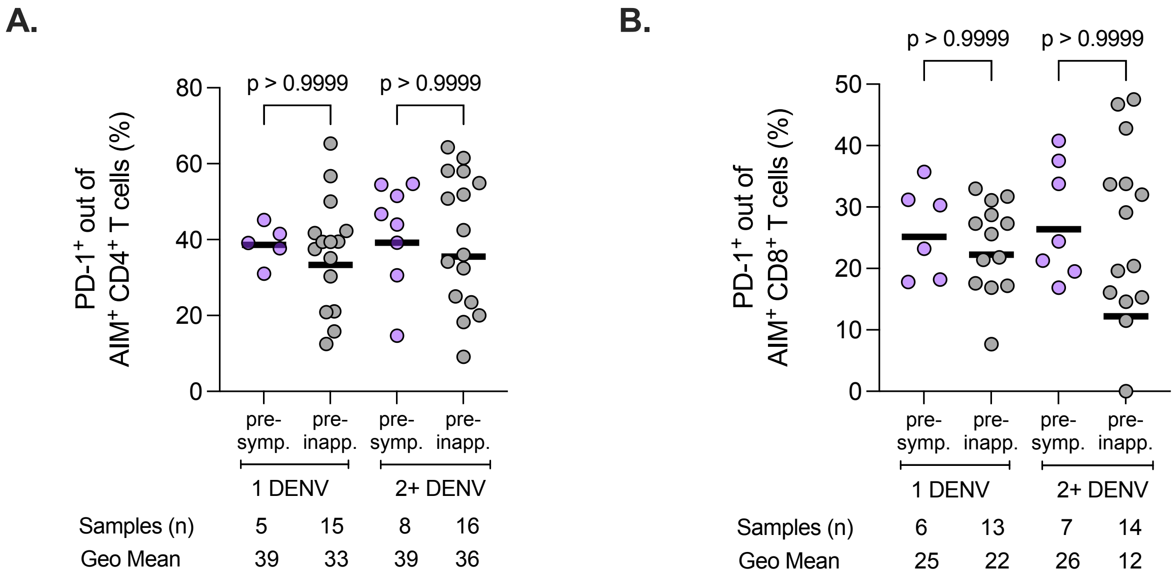

Figure S6. Expression of PD-1 within DENV-specific $CD4^+$ and $CD8^+$ T cell populations were stratified based on the number of prior DENV infections.

Table S1. Antibodies used for AIM assays

Marker	Fluorochrome	Clone	Source	Catalog. No.	Dilution
Live/Dead	Blue		ThermoFisher	L23105	1:1000
CCR6	BUV496	11A9	BD Biosciences	612948	1:200
CCR7	BV711	G043H7	Biolegend	353228	1:200
CD137	BUV737	4B4-1	BD Biosciences	741861	1:100
CD14	BV510	63D3	Biolegend	367124	1:1000
CD16	BV510	3G8	Biolegend	302048	1:1000
CD20	BV510	2H7	Biolegend	302340	1:1000
CD3	BUV395	UCHT1	BD Biosciences	563546	1:1000
CD38	BV650	HB-7	Biolegend	356620	1:200
CD4	cFluor b548	SK3	Cytek Biosciences	R7-20043	1:500
CD40L	PE-Dazzle594	24-31	Biolegend	310840	1:200
CD45RA	BV570	HI100	Biolegend	304132	1:1000
CD69	FITC	FN50	Biolegend	310904	1:200
CD8	BUV805	SK1	BD Biosciences	612889	1:1000
CD95	BB700	DX2	BD Biosciences	566542	1:500
CXCR3	BV605	G025H7	Biolegend	353728	1:200
CXCR5	BV421	J252D4	Biolegend	356920	1:200
HLA-DR	APC-R700	G46-6	BD Biosciences	565127	1:500
OX40	APC	Ber-Act35	Biolegend	350008	1:100
PD-1	BV785	EH12.2H7	Biolegend	329930	1:200

Table S2 HLA Phenotype and RATE Tool results for each donor.

Allele	A+R+	A+R+ %	No_of_Donors	Relative_freq	P-value
A*01:01	3	0.04	70	1.680	0.341
A*02:01	10	0.14	70	1.000	1.000
A*02:05	3	0.04	70	2.800	0.042
A*02:06	1	0.01	70	0.700	1.000
A*02:33	0	0	70	0.000	1.000
A*03:01	3	0.04	70	0.933	1.000
A*11:01	0	0	70	0.000	0.534
A*23:01	1	0.01	70	0.700	1.000
A*23:05	0	0	70	0.000	1.000
A*24:02	8	0.11	70	0.896	0.795
A*25:01	0	0	70	0.000	0.534
A*26:01	1	0.01	70	0.933	1.000
A*30:01	1	0.01	70	2.800	0.357
A*30:02	1	0.01	70	0.467	0.410
A*31:01	2	0.03	70	1.120	1.000
A*32:01	1	0.01	70	2.800	0.357
A*33:01	1	0.01	70	0.933	1.000
A*33:03	2	0.03	70	2.800	0.124
A*34:02	0	0	70	0.000	1.000
A*68:01	3	0.04	70	1.050	1.000
A*68:02	2	0.03	70	1.400	0.613
A*68:03	3	0.04	70	1.200	0.694
A*68:07	0	0	70	0.000	1.000
A*74:01	0	0	70	0.000	1.000
B*07:02	1	0.01	69	0.460	0.406
B*08:01	1	0.01	69	0.690	1.000
B*13:02	1	0.01	69	2.760	0.362
B*14:01	0	0	69	0.000	1.000
B*14:02	1	0.01	69	0.552	0.646
B*15:01	0	0	69	0.000	0.289
B*15:03	0	0	69	0.000	0.531
B*15:08	0	0	69	0.000	1.000
B*15:10	3	0.04	69	2.760	0.044
B*15:15	2	0.03	69	1.380	0.617
B*15:30	0	0	69	0.000	1.000
B*18:01	0	0	69	0.000	1.000
B*27:05	0	0	69	0.000	1.000
B*35:01	2	0.03	69	0.690	0.701
B*35:12	1	0.01	69	0.552	0.646
B*35:17	1	0.01	69	0.920	1.000
B*35:20	1	0.01	69	2.760	0.362
B*35:23	0	0	69	0.000	1.000
B*35:26	1	0.01	69	1.380	1.000

**B*35:43**	3	0.04	69	1.183	0.698
**B*38:01**	1	0.01	69	0.920	1.000
**B*39:02**	1	0.01	69	0.920	1.000
**B*39:05**	0	0	69	0.000	0.531
**B*39:08**	0	0	69	0.000	0.289
**B*39:11**	1	0.01	69	1.380	1.000
**B*40:01**	0	0	69	0.000	1.000
**B*40:02**	7	0.1	69	1.288	0.375
**B*40:04**	0	0	69	0.000	1.000
**B*40:11**	1	0.01	69	1.380	1.000
**B*41:01**	0	0	69	0.000	1.000
**B*41:02**	1	0.01	69	2.760	0.362
**B*44:02**	0	0	69	0.000	1.000
**B*44:03**	1	0.01	69	0.552	0.646
**B*45:01**	0	0	69	0.000	1.000
**B*49:01**	2	0.03	69	1.840	0.296
**B*50:01**	2	0.03	69	1.840	0.296
**B*51:01**	4	0.06	69	2.760	0.015
**B*52:01**	2	0.03	69	0.789	1.000
**B*53:01**	2	0.03	69	2.760	0.128
**B*56:01**	0	0	69	0.000	1.000
**B*57:01**	0	0	69	0.000	1.000
**B*58:01**	0	0	69	0.000	0.531
**C*01:02**	4	0.06	70	1.018	1.000
**C*02:02**	1	0.01	70	0.700	1.000
**C*02:10**	0	0	70	0.000	0.534
**C*03:02**	0	0	70	0.000	1.000
**C*03:03**	3	0.04	70	0.764	0.735
**C*03:04**	6	0.09	70	1.400	0.325
**C*03:05**	4	0.06	70	1.400	0.443
**C*04:01**	8	0.11	70	1.018	1.000
**C*05:01**	0	0	70	0.000	1.000
**C*06:02**	4	0.06	70	1.867	0.177
**C*07:01**	4	0.06	70	1.400	0.443
**C*07:02**	2	0.03	70	0.373	0.066
**C*07:05**	0	0	70	0.000	1.000
**C*07:41**	0	0	70	0.000	1.000
**C*08:02**	1	0.01	70	0.467	0.410
**C*12:03**	2	0.03	70	1.120	1.000
**C*15:02**	1	0.01	70	2.800	0.357
**C*16:01**	3	0.04	70	1.680	0.341
**C*17:01**	1	0.01	70	2.800	0.357
**DPB1*01:01**	3	0.04	70	1.200	0.694
**DPB1*02:01**	3	0.04	70	0.840	1.000
**DPB1*03:01**	3	0.04	70	0.840	1.000

**DPB1*04:01**	4	0.06	70	0.700	0.383
**DPB1*04:02**	15	0.21	70	0.933	0.611
**DPB1*05:01**	1	0.01	70	0.700	1.000
**DPB1*06:01**	1	0.01	70	2.800	0.357
**DPB1*10:01**	1	0.01	70	1.400	1.000
**DPB1*13:01**	1	0.01	70	0.700	1.000
**DPB1*14:01**	4	0.06	70	1.244	0.712
**DPB1*17:01**	2	0.03	70	1.120	1.000
**DPB1*18:01**	0	0	70	0.000	1.000
**DPB1*85:01**	1	0.01	70	2.800	0.357
**DQA1*01:01**	3	0.04	70	0.840	1.000
**DQA1*01:02**	4	0.06	70	1.018	1.000
**DQA1*01:03**	0	0	70	0.000	0.534
**DQA1*02:01**	3	0.04	70	2.100	0.127
**DQA1*03:01**	21	0.3	70	1.176	0.103
**DQA1*04:01**	2	0.03	70	0.431	0.116
**DQA1*05:01**	8	0.11	70	0.896	0.795
**DQB1*02:01**	1	0.01	69	0.690	1.000
**DQB1*02:02**	4	0.06	69	1.840	0.180
**DQB1*03:01**	8	0.12	69	1.227	0.409
**DQB1*03:02**	17	0.25	69	1.043	0.796
**DQB1*04:02**	4	0.06	69	0.649	0.256
**DQB1*05:01**	4	0.06	69	0.849	0.756
**DQB1*05:02**	1	0.01	69	1.380	1.000
**DQB1*05:03**	0	0	69	0.000	1.000
**DQB1*06:02**	2	0.03	69	0.789	1.000
**DQB1*06:03**	0	0	69	0.000	1.000
**DQB1*06:09**	0	0	69	0.000	0.531
**DRB1*01:01**	1	0.01	70	0.933	1.000
**DRB1*01:02**	1	0.01	70	1.400	1.000
**DRB1*01:03**	0	0	70	0.000	1.000
**DRB1*03:01**	1	0.01	70	0.700	1.000
**DRB1*03:02**	1	0.01	70	0.933	1.000
**DRB1*04:02**	1	0.01	70	0.933	1.000
**DRB1*04:03**	2	0.03	70	1.120	1.000
**DRB1*04:04**	2	0.03	70	1.120	1.000
**DRB1*04:05**	0	0	70	0.000	1.000
**DRB1*04:07**	13	0.19	70	1.174	0.452
**DRB1*04:10**	1	0.01	70	1.400	1.000
**DRB1*04:11**	2	0.03	70	1.120	1.000
**DRB1*04:17**	0	0	70	0.000	1.000
**DRB1*07:01**	4	0.06	70	2.240	0.051
**DRB1*08:02**	1	0.01	70	0.255	0.083
**DRB1*08:07**	0	0	70	0.000	1.000
**DRB1*09:01**	1	0.01	70	1.400	1.000

**DRB1*10:01**	0	0	70	0.000	0.534
**DRB1*11:01**	4	0.06	70	1.867	0.177
**DRB1*11:02**	1	0.01	70	2.800	0.357
**DRB1*11:04**	2	0.03	70	2.800	0.124
**DRB1*12:01**	0	0	70	0.000	0.289
**DRB1*13:01**	0	0	70	0.000	0.534
**DRB1*13:02**	0	0	70	0.000	0.289
**DRB1*13:03**	1	0.01	70	2.800	0.357
**DRB1*13:04**	2	0.03	70	1.867	0.289
**DRB1*14:01**	0	0	70	0.000	1.000
**DRB1*14:02**	2	0.03	70	1.400	0.613
**DRB1*14:06**	0	0	70	0.000	0.082
**DRB1*15:01**	1	0.01	70	0.700	1.000
**DRB1*15:03**	0	0	70	0.000	0.534
**DRB1*16:02**	1	0.01	70	2.800	0.357
**DRB3*01:01**	4	0.06	67	0.766	0.544
**DRB3*02:02**	9	0.13	67	1.269	0.401
**DRB3*03:01**	0	0	67	0.000	0.289
**DRB4*01:01**	21	0.31	67	1.082	0.381
**DRB4*01:03**	0	0	67	0.000	0.525
**DRB5*01:01**	1	0.01	67	0.536	0.643
**DRB5*02:02**	1	0.01	67	2.680	0.373
